# Psychological Resilience in Surgery: Psychobiological Pathways, Clinical Impact, and Perioperative Modulation—A Narrative Review

**DOI:** 10.3390/jpm16040178

**Published:** 2026-03-25

**Authors:** Giovanni Camardese, Marco Maria Pascale, Antonio Maria D’Onofrio, Rosaria Calia, Michele Ribolsi, Alexia Koukopoulos, Federico Fiori Nastro, Gaspare Filippo Ferrajoli, Elisa Schirra, Eleonora Maggio, Gabriele Sani, Gianluca Costa

**Affiliations:** 1 Department of Life Science, Health, and Health Professions, Link Campus University, 00165 Rome, Italy; 2 Department of Neuroscience, Head-Neck and Chest, Section of Psychiatry, Fondazione Policlinico Universitario Agostino Gemelli IRCCS, 00168 Rome, Italy; 3 Department of Surgery, Fondazione Policlinico Universitario Agostino Gemelli IRCCS, Università Cattolica del Sacro Cuore, 00168 Rome, Italy; 4 Department of Neuroscience, Section of Psychiatry, Università Cattolica del Sacro Cuore, 00168 Rome, Italy; 5 Unit of Clinical Psychology, Fondazione Policlinico Universitario Agostino Gemelli IRCCS, Università Cattolica del Sacro Cuore, 00168 Rome, Italy; 6 Department of Systems Medicine, Tor Vergata University of Rome, 00133 Rome, Italy; 7 IRCCS Fondazione Santa Lucia, 00179 Rome, Italy; 8 Department of Mental Health, ASL Latina, 04100 Latina, Italy; 9 Department of Translational Medicine and Surgery, Università Cattolica Del Sacro Cuore, 00168 Rome, Italy; 10 Clinical and Research Unit of Colorectal Surgery, Fondazione Policlinico Universitario Campus Bio-Medico, 00128 Rome, Italy

**Keywords:** resilience, perioperative care, surgical outcomes, mental health, surgical stress, personalized medicine

## Abstract

**Background and Objectives:** Psychological resilience is increasingly recognized as a determinant of how patients respond to surgical stress, yet its role in perioperative medicine remains poorly defined. This narrative review aims to synthesize current evidence on resilience in surgical populations from a psychobiological perspective, spanning conceptual models, measurement approaches, clinical correlates, biological mechanisms, and intervention strategies. **Materials and Methods:** This narrative review was conducted to examine psychological resilience in adult surgical populations from an integrated psychobiological and perioperative perspective. A structured literature search was performed in December 2026 using PubMed, Scopus, and PsycInfo, combining resilience-related constructs with surgical, perioperative, biological, and clinical outcome keywords. Eligible publications included observational, longitudinal, interventional, translational, and conceptually relevant studies addressing resilience in adult surgical settings. Evidence was synthesized qualitatively across predefined domains, including conceptualization and measurement of resilience, associations with perioperative outcomes, neuroendocrine and inflammatory mechanisms, and resilience-modulating interventions within perioperative and Enhanced Recovery After Surgery (ERAS) frameworks. **Results:** Contemporary models conceptualize resilience as a dynamic, context-dependent process supported by interacting psychological, biological, and social factors. In surgical cohorts, higher resilience is consistently associated with better patient-reported outcomes, including quality of life, pain control, and emotional adjustment, and in some studies with survival and functional recovery. Preoperative depression, anxiety, maladaptive coping, and low social support converge as components of a broader “resilience profile” linked to poorer postoperative trajectories. Biologically, resilient phenotypes are characterized by more regulated hypothalamic–pituitary–adrenal and autonomic responses and reduced inflammatory activation. Psychological therapies, prehabilitation programs, and selected pharmacological strategies show convergent, though heterogeneous, signals of benefit and can be interpreted as indirect resilience-enhancing interventions. **Conclusions:** Resilience appears to be a clinically meaningful, potentially modifiable construct that links psychosocial functioning, biological vulnerability, and postoperative outcomes. Incorporating resilience assessment into preoperative risk stratification and systematically embedding resilience-building strategies within perioperative and ERAS pathways may support more personalized, psychologically informed surgical care. Prospective, multidomain studies are needed to validate measurement tools, clarify mechanisms, and test resilience-targeted interventions in surgical populations.

## 1. Introduction

Resilience has gained increasing attention within clinical and behavioral sciences, emerging as a key determinant of how individuals respond to stress, illness, and recovery. The perioperative setting represents a unique and controlled model of stress exposure, in which physiological, psychological, and social systems are simultaneously challenged.

The aim of this narrative review is to synthesize current evidence on resilience in surgical populations through a psychobiological lens. In doing so, we first examine the principal theoretical frameworks that define resilience from both psychological and neurobiological perspectives. We then address the methodological tools used to assess resilience, focusing on the instruments most employed in research. Subsequently, we review the evidence linking preoperative psychological functioning and psychosocial determinants—such as anxiety, depression, coping strategies and social support—to postoperative trajectories. Building on this, we explore the biological mechanisms that may underlie resilient recovery, considering neuroendocrine, autonomic and immunological pathways that contribute to individual variability in surgical outcomes. Finally, we discuss current and emerging interventions aimed at enhancing resilience before and after surgery, including psychological therapies, behavioral programs, pharmacological agents and multimodal prehabilitation strategies integrated within perioperative care.

Through this integrative approach, the review seeks to elucidate the clinical relevance of resilience in perioperative medicine and to consider its potential inclusion in risk stratification models and personalized surgical care pathways.

## 2. Materials and Methods

This manuscript was designed as a narrative review to examine psychological resilience in adult surgical populations from an integrated psychobiological and perioperative perspective. Given the conceptual complexity of resilience and the multidisciplinary scope of perioperative research, a narrative approach was selected to allow theoretical integration and cross-domain synthesis rather than formal systematic aggregation. Accordingly, this review was not intended to be exhaustive or to follow systematic review reporting standards, but rather to provide a conceptually driven and integrative synthesis of the literature.

A structured literature search was performed in January 2026 using PubMed, Scopus, and PsycInfo. Search terms combined resilience-related constructs (“resilience,” “psychological resilience,” “stress adaptation,” “coping”) with perioperative and surgical keywords (“surgery,” “perioperative,” “preoperative,” “postoperative,” “anesthesia,” “enhanced recovery,” “ERAS,” “prehabilitation,” “frailty”). Additional terms targeted biological mechanisms (“hypothalamic–pituitary–adrenal axis,” “autonomic nervous system,” “inflammation,” “cytokines,” “neuroendocrine”) and clinical outcomes (“pain,” “quality of life,” “functional recovery,” “complications,” “mortality”). Search results were screened for thematic relevance by the authors, with emphasis on studies that contributed to the conceptual framework of resilience in surgical settings rather than on comprehensive coverage of all available evidence.

Eligible publications included original observational studies, longitudinal cohorts, interventional trials, and translational investigations addressing resilience or closely related constructs in adult surgical settings. Conceptual and theoretical contributions were included when directly relevant to perioperative stress adaptation. Studies exclusively focused on non-surgical populations were considered only if they provided mechanistic insights applicable to surgical stress physiology. Systematic reviews and meta-analyses were used to contextualize findings but were not treated as primary data sources. Given the narrative design, study inclusion was guided by relevance to the predefined conceptual domains, and some potentially relevant studies may not have been included if they did not directly align with the thematic focus of the review.

Articles were limited to those published in English. Study selection was guided by predefined thematic domains: (1) conceptualization and measurement of resilience; (2) associations between resilience and perioperative outcomes; (3) neuroendocrine, autonomic, and inflammatory correlates; and (4) resilience-modulating interventions embedded within perioperative care pathways, including ERAS frameworks.

Data were synthesized qualitatively through thematic integration to identify convergent evidence, mechanistic hypotheses, and translational implications. In line with the narrative design, no formal risk-of-bias assessment or meta-analytic procedures were conducted. This methodological approach reflects the exploratory and hypothesis-generating aim of the review rather than a quantitative evidence appraisal.

## 3. Conceptual Framework of Resilience

### 3.1. Psychological Theories and Models

The construct of resilience has its roots in developmental psychology [1,2,3,4]. However, starting from the early 2000s, research into resilience has become increasingly popular and has been examined across numerous populations and domains [2,5,6]. Despite this substantial growth, the proliferation of definitions and theoretical frameworks has raised concerns regarding the conceptual clarity of the term [7]. For example, an examination of mental health publications from the World Health Organization (WHO) illustrates the lack of a shared, consistently applied definition [8].

Resilience is commonly described as the capacity to withstand, adapt to, and recover from adversity or stress, although the degree to which individuals demonstrate such adaptive responses varies considerably [8]. Importantly, not all stress responses are pathological; some represent functional coping strategies that contribute to psychological adjustment.

Early conceptualizations of resilience framed it as a stable personality trait [9]. Research in this tradition focused on identifying children who, despite growing up in adverse conditions, were able to achieve positive developmental outcomes. This perspective assumed that individuals possessed innate, context-independent characteristics that enabled them to resist or overcome stressors. Accordingly, children who scored highly on measures of trait resilience were presumed to demonstrate adaptive functioning across situations and over time, regardless of the nature of environmental demands or their prior exposure to stress. Within this framework, resilience was viewed as an enduring, static attribute that shaped consistent patterns of behavior and coping (e.g., problem-focused strategies). Trait resilience has been associated with characteristics such as self-efficacy, humor, and an internal locus of control [10,11]. However, the foundational assumptions of trait-based models have been increasingly challenged [12].

More recent models emphasize dynamic, context-dependent processes rather than fixed traits. Rutter [13], for instance, proposed that resilience emerges through the interaction between individual characteristics and environmental conditions. According to this perspective, protective factors influence how a person responds to potential stressors, such that adverse outcomes may be mitigated. Notably, these protective effects often become apparent only when individuals are confronted with significant challenges [14].

Empirical evidence supports the idea that resilience is not fixed but instead fluctuates across the lifespan, shaped by developmental, psychological, and contextual factors [15]. Moreover, adaptive responses to adversity appear to be history-dependent: the impact of a stressor is influenced by prior life experiences and the broader environmental context in which the stressor occurs [16,17]. Further, individuals may also require varying amounts of time to recover, particularly when multiple stressors occur in close succession.

Increasingly, resilience is conceptualized not as a fixed attribute, but as a dynamic process of adjustment to change and adversity [18]. Studies adopting this approach have demonstrated that resilience assessed as a stable trait does not consistently predict changes in well-being, whereas models that capture resilience as an unfolding process reveal substantial adaptive variability [19]. Similarly, longitudinal evidence indicates that increases in resilience can precede reductions in psychological symptoms, supporting a temporal, process-based understanding [20]. Dynamic models also permit the identification of non-linear trajectories of change that trait models cannot capture [21]. Furthermore, resilience is now increasingly understood as a network of interacting protective factors rather than a single individual characteristic [22]. Collectively, these developments reflect a conceptual shift from viewing resilience as an inherent, stable personality trait to regarding it as a complex, adaptive, context-dependent process that unfolds over time in response to adversity.

### 3.2. Neurobiological Underpinnings

An expanding body of literature has investigated the principal physiological stress-response cascade, commonly referred to as the hypothalamic–pituitary–adrenal (HPA) axis [23]. Among the primary molecular mediators of the stress cascade are the hypothalamic corticotropin-releasing factor (CRF), which constitutes the final common pathway integrating neural signals that initiate the peripheral endocrine response to stress, and adrenocorticotropic hormone (ACTH), which, upon CRF stimulation, is secreted from the anterior pituitary into systemic circulation. ACTH subsequently acts on the adrenal cortex to promote the synthesis and release of glucocorticoid stress hormones such as cortisol in humans [24].

Circulating glucocorticoids exert their effects through two principal steroid hormone receptors: the glucocorticoid receptor (GR) and the mineralocorticoid receptor (MR). These receptors are expressed in cells across most organ systems, where they regulate glucose metabolism, energy expenditure, and the overall physiological response to stress. Notably, both GR and MR are also expressed within the brain [25]. At a broad physiological level, the activation of GRs within the hippocampus and cerebral cortex functions as an indicator of elevated circulating stress steroid concentrations, thereby triggering negative feedback mechanisms that attenuate and ultimately terminate the endocrine stress response [26,27]. Both GRs and MRs are integral components of complex signaling networks that have been implicated in the pathophysiology of depression and other mood disorders. The majority of human studies investigating the effects of stress on HPA axis functioning have primarily focused on Major Depressive Disorder (MDD) and Post-Traumatic Stress Disorder (PTSD) [28].

The neural circuits underlying stress regulation involve two distinct yet interacting mechanisms: bottom-up and top-down processes [29]. Bottom-up processes are generally considered automatic and reactive, as they encompass cognitive and emotional responses that are reflexively triggered by emotionally salient stimuli arising from, or activated within, subcortical structures [30]. In contrast, top-down processes entail deliberate and reflective cognitive control that engages higher-order cortical regions. Empirical evidence indicates that bottom-up and top-down mechanisms are functionally distinct, recruit different cognitive and emotional regulatory systems, and interact in a bidirectional manner [31].

Neural pathways originating in subcortical limbic structures—such as the hypothalamus and amygdala—modulate the activity of higher cortical regions through projections to the medial prefrontal cortex [32]. This activation contributes to the emergence of self-referential and repetitive thought patterns, forming the basis for the sustained processing of negative information within working memory [33]. The bidirectional connectivity between limbic structures, particularly the amygdala, and the medial prefrontal cortex may facilitate the amplification and prolongation of negative emotional responses [34]. Collectively, two primary pathways can be identified. The first links the amygdala with the hippocampus, caudate nucleus, and putamen. The second involves the activation of a higher-order cortical structure—the right dorsolateral prefrontal cortex—which plays a pivotal role in anticipating negative emotional stimuli by allocating attentional and cognitive resources toward them [34]. This region exerts top-down regulatory control over emotional processing, attenuating distress signals, for instance, by dampening the HPA axis response to stress.

### 3.3. Measurement Approaches

The early conceptualization of resilience as a fixed trait led to the widespread use of standardized, cross-sectional questionnaires in clinical research to estimate an individual’s likelihood of developing psychological difficulties [9,35]. Particularly, resilience in surgical populations has been commonly assessed using self-report psychometric instruments such as the Connor-Davidson Resilience Scale (CD-RISC) [36], the Brief Resilience Scale (BRS) [37], and the Resilience Scale for Adults (RSA) [38]. These tools aim to quantify perceived coping resources, adaptability, and recovery capacity and have shown acceptable internal consistency and construct validity in general clinical settings.

The BRS, which consists of six Likert-type items, was developed to assess resilience in terms of health-related outcomes and an individual’s capacity to “bounce back” from stress. Its developers reported strong test–retest reliability and good internal consistency. Subsequent studies further supported its reliability and have demonstrated its applicability across diverse research settings and populations, including both healthy individuals and those with mental health conditions [39]. The BRS has been utilized in various international contexts, including the United States, Spain, China, and Brazil. However, the scale has been criticized for lacking a systematic procedure to modify items that do not translate effectively into non-English languages. As a result, concerns have been raised regarding its cultural adaptability and its suitability for use across diverse linguistic and cultural settings [39,40,41].

The CD-RISC-25 is a 25-item Likert-type instrument developed in 2003 to assess an individual’s capacity to cope with stress [4,36]. The scale was designed to demonstrate that resilience is a modifiable construct that can improve following targeted intervention, implying that recovery from stressful experiences may be enhanced through resilience training [36,42]. The scale and its population-specific adaptations have shown cross-cultural validity and have been applied effectively in diverse groups, including elite athletes, military personnel, surgical patients, and trauma survivors [43,44,45]. However, several limitations have been noted. The authors themselves acknowledge that the CD-RISC-25 has not yet been validated against objective physiological indicators of stress, such as measurable neurobiological responses [36]. Additionally, the scale does not capture domain-specific variability in resilience; an individual may demonstrate high resilience in one context (e.g., occupational stress) while simultaneously exhibiting low resilience in another (e.g., interpersonal or family stress), a nuance not reflected in the overall score [36].

The RS, developed in 1993, is a 25-item instrument designed to assess resilience across five dimensions: equanimity, perseverance, meaningfulness, self-reliance, and existential aloneness [46]. The RS was developed to evaluate resilience in the context of an individual’s adaptive responses to life challenges. It is one of the most widely utilized resilience measures and has been applied across a broad age range, from adolescents to older adults [47]. While the scale demonstrates acceptable validity and internal consistency, its reproducibility (test–retest reliability) has been reported as comparatively lower than that of other commonly used instruments such as the BRS and the CD-RISC-25 [47].

A comprehensive review of surgical research demonstrated that the BRS (41.9% of studies) and the CD-RISC (29.7% of studies) were the most frequently used measures, both of which confirmed strong psychometric properties [48]. However, their applicability to surgical contexts remains debated. Surgical stressors are highly specific, encompassing physical trauma, postoperative pain, anesthesia effects, and psychosocial concerns related to recovery and bodily integrity, and standard resilience scales may not fully capture these situational demands. Additionally, assessing resilience preoperatively presents several challenges: patients may respond under acute anxiety or uncertainty, leading to unstable or inflated scores, and the self-report nature of most available measures inherently captures resilience at a single time point. Consequently, these assessments tend to reflect more trait-like perceptions rather than the dynamic and context-dependent processes that characterize resilience during recovery. Therefore, longitudinal and repeated assessments are recommended to better capture fluctuations over time. Furthermore, study samples should be expanded to include more diverse surgical populations to more conclusively determine the validity and clinical utility of these instruments. Consequently, while psychometric tools can provide useful baseline information, they suffer from limitations [48] including inconsistent definitions of resilience, predominantly single-center studies, and generally low-quality evidence and they should be interpreted cautiously and ideally supplemented with longitudinal or behavioral assessments to more accurately evaluate resilience in surgical patients.

## 4. Psychosocial Determinants and Surgical Outcomes

### 4.1. Preoperative Psychological Predictors

A robust literature demonstrates that preoperative psychological functioning exerts a significant and independent influence on postoperative trajectories across surgical specialties. In particular, preoperative depression has been consistently associated with an increased risk of postoperative delirium and a broader spectrum of adverse outcomes. Depressed patients appear more vulnerable to thromboembolic events, infectious and neurological complications, hemodynamic instability, and urinary disturbances, as well as higher rates of readmission, reoperation, and non-routine discharge, even when hospitalization length does not markedly differ from non-depressed controls [49,50]. Similarly, greater severity of depressive and anxiety symptoms has been linked to an elevated likelihood of postoperative complications, further underscoring the clinical relevance of preoperative psychological assessment [51].

Similarly, preoperative anxiety functions as a potent risk factor for adverse perioperative outcomes. Meta-analytic evidence indicates that higher levels of preoperative anxiety are associated with increased anesthetic and analgesic requirements, as reflected by significant standardized mean differences in intraoperative drug consumption and postoperative pain management [52]. Preoperative anxiety has also been linked to an elevated risk of postoperative delirium in adults, although this association has not been consistently observed in pediatric populations [52]. In addition, anxious patients demonstrate prolonged recovery parameters, including delayed attainment of a Modified Aldrete Score ≥ 9 and longer extubation times, as well as greater propofol consumption during anesthesia [52]. In cardiac surgery, each incremental increase in preoperative state anxiety has been associated with higher postoperative morphine requirements, whereas advancing age appears to exert a protective effect by reducing analgesic needs; no significant sex differences have been observed in this regard [53]. Although preoperative pain levels do not consistently predict postoperative pain, preoperative anxiety shows a moderate positive correlation with postoperative pain intensity [54]. Furthermore, anxiety is closely intertwined with cognitive–emotional constructs such as pain catastrophizing and optimism. Pain catastrophizing, in particular, has been identified as a significant predictor of anticipated postoperative pain and fully mediates the relationship between anxiety and anticipated pain outcomes, accounting for a substantial proportion of variance in postoperative pain expectations [55].

Coping style represents another critical determinant of postoperative trajectories. Patients experiencing complicated recovery courses have been shown to report reduced life satisfaction and lower situation-specific self-control expectations, with structural equation modeling demonstrating direct associations between surgical recovery and personality dimensions such as life satisfaction, extraversion, and attainment orientation [56]. Coping strategies adopted in the perioperative period further modulate psychological adjustment and distress. Approach-oriented strategies, including cognitive processing and support seeking, appear particularly relevant in reducing postoperative distress, whereas certain forms of detachment may exert variable effects depending on context [57]. In orthopedic populations, improvements in functional outcomes have been closely linked to adaptive cognitive coping mechanisms, with coping self-statements mediating the relationship between pain interference and functional recovery [58]. Qualitative evidence similarly highlights the importance of adaptive coping styles—such as positive reframing, optimism, proactive engagement, and humor—while identifying passive acceptance, avoidance, and denial as maladaptive patterns associated with poorer psychological adjustment [59]. Consistently, problem-focused coping strategies have been positively associated with improved surgical outcomes, whereas emotion-focused coping does not demonstrate the same predictive value in some surgical contexts [60].

Prospective data from orthopedic trauma populations further reinforce the prognostic relevance of early psychological factors. Within one week of injury, greater pain interference, pain catastrophizing, fear of movement, and lower self-efficacy significantly predicted functional limitations at 6–9 months, even after controlling for injury severity and demographic variables [61]. Similarly, lower social and instrumental support and higher anxiety symptoms were independently associated with prolonged opioid use over time [62]. In longitudinal models, catastrophic thinking emerged as the strongest predictor of persistent pain and disability, explaining a substantial proportion of variance beyond injury-related factors [63].

Converging evidence also highlights the importance of perceived social support, which consistently emerges as a robust protective factor across surgical populations. Emotional, informational, and instrumental support modulate stress responses, improve adherence to physiotherapy, and reduce postoperative distress. Socially supported patients report lower pain, fewer complications, and faster return to daily functioning, particularly in cardiac and oncologic surgery, where postoperative needs are substantial [64,65]. Mechanistically, social support enhances emotion regulation, reduces systemic stress reactivity, and sustains motivation during prolonged rehabilitation pathways.

In our view, resilience may represent the underlying construct that connects psychosocial functioning with postoperative recovery. Rather than considering depression, anxiety, coping style, or social support as separate predictors, these factors can be interpreted as interconnected components of a broader resilience profile that shapes how patients respond to surgical stress. This perspective might not only clarify interindividual variability in recovery trajectories, but also guide the development of perioperative strategies aimed at proactively strengthening patients’ adaptive resources. In this sense, resilience could represent a clinically meaningful framework through which to interpret vulnerability and promote recovery in surgical populations.

### 4.2. Resilience as a Protective Moderator

Across surgical fields, resilience functions as a powerful moderator of postoperative outcomes. In oncology, higher resilience has been associated with reduced perioperative distress, more effective symptom management, and improved long-term quality of life following procedures such as mastectomy, breast reconstruction, and major abdominal cancer surgery [48,66].

In cardiac surgery, elevated resilience scores predict lower levels of postoperative anxiety and depression, enhanced adherence to cardiac rehabilitation, and more rapid recovery of functional capacity after coronary artery bypass or valvular procedures. Emerging work also suggests that psychological resilience may attenuate inflammatory responses and autonomic dysregulation, yielding downstream benefits in physical recovery and symptom burden [67,68,69,70].

In orthopedic surgery, particularly joint replacement and spine procedures, resilience has been shown to predict postoperative pain tolerance, accelerated return to mobility, and greater satisfaction with surgical outcomes. Several studies indicate that resilience is a stronger predictor of functional recovery than demographic variables or baseline pain levels, emphasizing its unique contribution to rehabilitation potential. Resilience appears to influence not only immediate postoperative outcomes but also longer-term physical and psychosocial adjustment [71,72,73,74]. Additional evidence demonstrates similar patterns in less invasive procedures such as arthroscopic rotator cuff repair, with resilience functioning as a significant predictor of pain reduction, sleep quality, and patient-reported outcomes at two years [75,76].

In bariatric surgery, higher resilience is associated with superior adherence to dietary and behavioral recommendations, lower postoperative emotional distress, and more stable weight trajectories. Resilient patients demonstrate more adaptive responses to profound body-image changes, reduced vulnerability to depression, and improved psychological adjustment during the intensive lifestyle modifications required after surgery [77,78,79].

Across all surgical contexts, resilience mitigates the negative effects of preoperative depression, anxiety, and maladaptive coping. It may act as a psychological buffer that enhances cognitive flexibility, emotional regulation, physiological stress recovery, and engagement in rehabilitation, thereby optimizing postoperative outcomes.

### 4.3. Interaction with Frailty and Comorbidity

Resilience must also be conceptualized in relation to frailty, medical comorbidity, and the broader biopsychosocial vulnerability profile of surgical patients. Frailty—characterized by diminished physiological reserve, reduced homeostatic capacity, and increased inflammatory reactivity—substantially elevates the risk of postoperative complications, delirium, and functional decline. Notably, resilience has been shown to partially counterbalance these risks by promoting emotional regulation, motivational engagement, and adherence to postoperative care protocols, thus moderating the impact of biological vulnerability on surgical outcomes [80,81].

Innovative studies employing multidomain measures of resilience—including neuroendocrine, autonomic, and cognitive-motor components—demonstrate that lower resilience is associated with increased frailty, higher postoperative complication rates, and greater deterioration in one-year quality of life among older surgical patients. In high-risk procedures such as pancreatectomy, resilience appears to influence not only short-term recovery but also longer-term functional trajectories and vulnerability to postoperative decline [82].

Within contemporary biopsychosocial models, resilience is understood as an integrative construct that connects a patient’s psychological resources with biological factors (frailty phenotype, comorbidity burden, inflammatory status) and social determinants (support networks, socioeconomic conditions). High resilience may attenuate the functional impact of comorbid conditions—such as diabetes, cardiovascular disease, or chronic pain—on surgical recovery by enhancing engagement in postoperative care, strengthening self-management behaviors, and modulating stress-related neuroendocrine pathways [83,84].

Conceptually, resilience operates as a dynamic moderator that shapes how frailty and comorbidity translate into postoperative complications or prolonged recovery. Assessing resilience preoperatively may therefore improve risk stratification, inform personalized perioperative psychological interventions, and ultimately contribute to more patient-centered and successful surgical outcomes.

## 5. Biological Mechanisms Linking Resilience and Recovery

### 5.1. Neuroendocrine Stress Response

The two major branches of the autonomic nervous system (ANS) are the sympathetic nervous system (SNS), involved in the fight-or-flight response, and the parasympathetic nervous system (PNS), involved in vegetative functions and the rest-and-digest response [85]. These two systems are in balance in the normal state; however, external stressors can create a disruption leading to an imbalance towards a hyperactivation of the sympathetic nervous system, which in turn creates a disruption of the HPA axis. The HPA axis consists of a system of positive stimulatory signals and negative feedback loops, ultimately leading to glucocorticoid production by the adrenal medulla [86]. Glucocorticoids are steroid hormones involved in a plethora of different mechanisms, such as regulation of the inflammatory and immune response, cardiovascular tone, metabolism, growth and cognitive functions [86]. In a normal state, cortisol, the most commonly employed biomarker produced by the HPA axis, follows a circadian rhythm: its levels are higher in the morning and gradually drop until they reach a nadir in the evening [87]. External stressors are able to disrupt normal HPA axis functioning, as they stimulate corticotropin-releasing hormone secretion in the hypothalamus, which in turn activates the axis cascade, ultimately leading to a dysregulated glucocorticoid release [88].

An alteration of the cortisol circadian rhythm has been shown to be associated with worse mental and physical health and even to an increase in cancer and mortality [87]. Moreover, the circadian rhythm allows a balance between resilience and vulnerability towards external stressors, as it can allow some degree of anticipation of daily challenges (for instance, stimulating the body activation in the morning and promoting rest in the night); on the other hand an imbalance in this pathway could lead to an increased vulnerability and reduced resilience, associated with abnormalities in cognitive and physiological functions [88].

### 5.2. Immune and Inflammatory Regulation

An inflammatory reaction in the body typically involves signaling proteins, i.e., cytokines and acute phase proteins, which coordinate the immune response to the trigger. These proteins are often used as inflammatory biomarkers, the most important and commonly used being Interleukins (IL-1β, IL-17 and IL-6), Tumor Necrosis Factor Alpha (TNF-α) and C-reactive Protein (CRP) [89]. Inflammatory cytokines recruit white blood cells targeting pathogens, while activation of the complement system, involving acute phase proteins, directly targets the cell membrane [89]. The HPA axis is influenced both by these proteins and by sympathetic system activation, causing cortisol release: this is thought to have at first a pro-inflammatory and then an anti-inflammatory function [89].

These biomarkers have been studied in individuals in high-stress environments: a study by Wong et al. [90] has shown that total resilience scores (measured with Hardiness Resilience Gauge) are inversely correlated with salivary levels of IL-6 and TNF-α during stress exposure, possibly suggesting that resilient phenotypes have a reduced cytokine surge and thus mount a less pronounced inflammatory response. A study by Yaseen et al. [91] reports that patients with lower preoperative anxiety (which can be interpreted as a marker for higher resilience) showed lower proinflammatory cytokine (IL-1b, IL-6, TNF-α and IL-2) levels compared to those who manifested anxiety. Anxious patients were also shown to have poorer postoperative outcomes and a higher incidence of complications and leukocytosis compared to the non-anxious group. Moreover, Imai et al. [92] found that individuals with lower resilience and lower perceived quality of life had higher IL-6 and CRP values.

These findings support a relation between a resilient phenotype and a better modulated immune response, linked to reduced systemic inflammation.

### 5.3. Autonomic and Metabolic Correlates

The vagus nerve is a key component of the parasympathetic nervous system, regulating neurovegetative functions such as cardiac activity, which can be monitored via heart rate variability (HRV) [93,94]. It is also involved in the so-called inflammatory reflex, which can down-regulate peripheral inflammation [94]. Considering the important role of the vagus nerve, evaluating its activity, especially in the perioperative setting, can provide relevant information to optimize patients’ care. The HRV can be measured by observing respiratory modulation of cardiac arrhythmia, as the vagus increases heart rate during inspiration and slows it down during expiration [94]. It has therefore been observed that HRV is negatively impacted by surgery, leading to a hindered inflammatory reflex: both this scenario and surgery itself lead to an increased inflammation [94]. It is therefore important to preserve vagal function to enhance recovery: in fact, individuals with a higher preoperative HRV (which correlates to an increased vagal tone) were found to have improved outcomes, an increased and faster benefit from post-operative rehabilitation, with an increased variability in the weeks following surgery [93,94].

It has been shown that psychological factors can negatively impact HRV: major depressive disorder and post-stress behaviors (such as rumination) were associated with an impaired recovery after exposure to a stressor, while individuals with greater resilience and higher oxytocin levels had a greater rehabilitation [95].

These observations could therefore provide an additional perspective to take into consideration for an enhanced recovery after a stressor such as surgery.

### 5.4. Psychoneuroimmunology of Wound Healing and Recovery

Recent literature reports that psychological resilience is associated with an improved surgical recovery, including better patient-reported outcomes (such as a higher quality of life, greater satisfaction, better mental health and reduced pain) and an improved functional outcome [48]. Moreover, a study on a cohort of older adults has shown that a stronger resilience was associated with a decreased risk of 2.5-year postoperative mortality and a lower risk of decline in instrumental activities of daily living; in turn, individuals with depression were at higher risk [96]. This observation could be attributed to the adaptive response to stress in resilient individuals, who exhibit a more controlled cortisol response, limiting systemic inflammation. While it is difficult to have definite results due to variations in patient population and to the complexity of assessing an individual’s resilience, these studies give a promising perspective on a possible preoperative intervention: improving resilience could in fact have a tangible impact on surgical outcome. Indeed, psychological prehabilitation (by means of cognitive behavioral therapy, supportive psychotherapy and acceptance and commitment therapy) has been shown to reduce length of hospital stay, pain, anxiety and depression after surgery [97].

**Figure 1:**
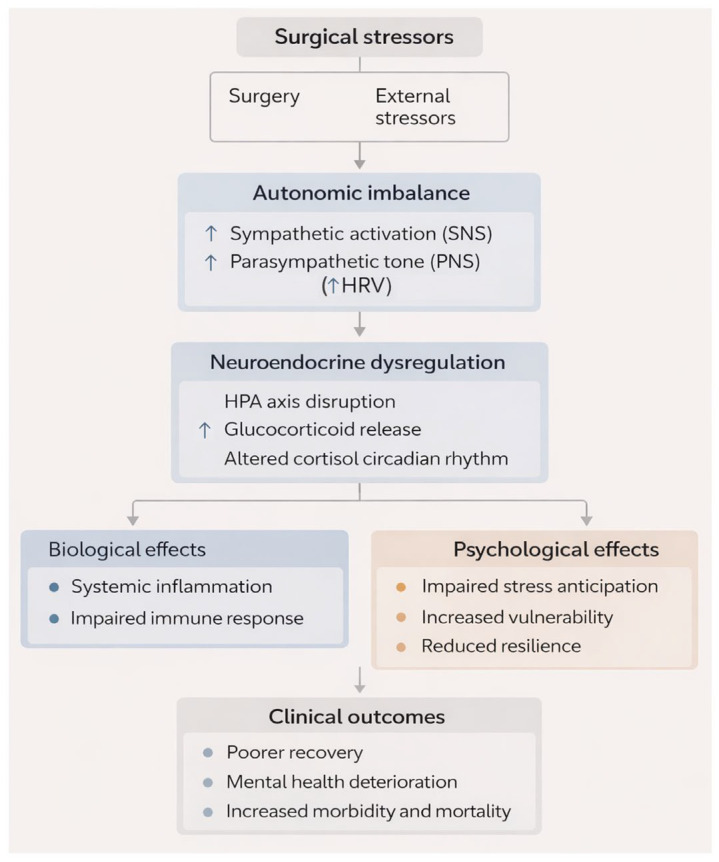
Psychobiological pathways linking surgical stress to autonomic dysregulation, inflammation, and reduced resilience. HRV: Heart Rate Variability.

To integrate the psychobiological mechanisms discussed above, we propose a conceptual model summarizing how surgical stress may translate into recovery trajectories (Figure 1).

## 6. Interventions to Enhance Resilience Before and After Surgery

### 6.1. Psychological and Behavioral Strategies

An increasingly refined body of evidence indicates that psychological variables can meaningfully influence the trajectory of postoperative recovery. For this reason, psychological and behavioral procedures have been integrated into protocols for both postsurgical recovery and physical rehabilitation. Specifically, targeted interventions such as cognitive-behavioral therapy (CBT), mindfulness-based techniques, and relaxation training have been employed. Collectively, these approaches adopt a biopsychosocial framework and show significant signals of efficacy in improving postoperative outcomes. Notably, the literature highlights that psychological strategies enhance self-efficacy and favorably shape individual resilience, including its impact on preoperative anxiety. Prospective evidence demonstrates that preoperative resilience is an independent predictor of superior functional outcomes. In the REGAIN trial, higher resilience scores were associated with reduced odds of the composite endpoint “death or new inability to walk” at 60 days (aOR 0.77; 95% CI 0.61–0.98), with a more pronounced effect in patients without postoperative complications [98].

According to the international literature, psychological techniques modulate the psychoneuroendocrine and immune systems, mitigating maladaptive distress responses and, in some cases, decreasing inflammatory markers [99]. Interventions targeting preoperative anxiety through relaxation techniques and mindfulness have been associated with reductions in pain catastrophizing prior to surgery and with improved pain outcomes postoperatively. Buvanendran and colleagues observed reductions in catastrophizing in a randomized study of patients undergoing knee arthroplasty who received preoperative CBT programs delivered either in person or remotely. Although improvements in pain at three months did not reach statistical significance, the authors hypothesized that therapeutic benefit may require further refinement and optimized timing [100].

In spine surgery, a systematic review suggested that perioperative CBT may reduce pain and disability in the short term, although evidence was less robust at medium- and long-term follow-up [101]. To prevent the transition from acute to chronic pain, ongoing trials are evaluating CBT interventions in adolescents at risk (e.g., following spinal fusion), with the aim of interrupting the catastrophizing–avoidance–disability cycle [102]. A pilot study by Hadlandsmyth et al. 2022 examined a perioperative pain self-management program in adults, demonstrating feasibility and preliminary efficacy in reducing emotional distress. The authors propose that early intervention may shape long-term pain trajectories and opioid use [103].

Mindfulness-based interventions—aimed at reducing preoperative anxiety and postoperative pain—have been associated with greater pain tolerance and reduced anxiety, with benefits extending to physical functioning at six weeks [104]. A meta-analysis by Tung and colleagues demonstrated clear reductions in preoperative anxiety and postoperative pain in patients exposed to mindfulness at multiple timepoints: immediately after intervention, and at 2–3 days, 14 days, and 28 days [105]. A parallel line of research has examined mindfulness delivered through virtual reality (VR), comparing it with other strategies such as music therapy across different surgical contexts. Preliminary findings on anxiety before and after surgery are promising but limited by small sample sizes. In preoperative VR studies, control groups typically received standard care or non-musical distraction tasks. Meta-analyses of VR predominantly synthesize comparisons with standard care [106], whereas aggregated evidence on music therapy derives from dedicated reviews [107]. Overall, effects on anxiety and pain are encouraging yet heterogeneous; comparisons with music therapy arise from separate, methodologically diverse studies with limited power.

Work by Van der Horst and colleagues on Acceptance and Commitment Therapy (ACT), which aims to increase psychological flexibility and pain tolerance, suggests that ACT aligns well with the surgical context—characterized by high acute stress and diminished perceived controllability. Among patients undergoing spine surgery, a digital intervention combining ACT with positive psychology (“Strength Back”) yielded, in a non-randomized pilot study, greater improvements in emotional and overall well-being and a more marked reduction in pain intensity compared with controls. Adherence to program modules was high and acceptability favorable, paving the way for confirmatory randomized trials [108]. In breast cancer surgery, an ongoing randomized trial combines preoperative hypnosis with postoperative web-based ACT to prevent persistent pain and fatigue and reduce work absenteeism. The rationale integrates acute phase prevention (hypnosis) with long-term rehabilitation (ACT), including the collection of clinical outcomes and biomarkers up to 12–24 months [109].

A new generation of mental skills training and “stress vaccine” strategies has also been tested in high-intensity clinical settings such as Emergency Departments, operating rooms, and intensive care units, using simulations designed to elicit strong emotional pressure. Evidence from short-term trials is mixed (for example, no clear benefit on stress or performance in a randomized study in Emergency Medicine residents), but the model remains promising and potentially transferable to perioperative psychological prehabilitation. Such approaches incorporate educational components, coping skills, and imaginative exposure to perioperative stressors [110].

These strategies operate through shared mechanisms that influence recovery: they reduce catastrophizing and recalibrate pain expectations, enhance self-efficacy and emotion regulation capacity, increase psychological flexibility (ACT), and promote proactive coping through graded exposure to stressors. The mastery of these techniques—before being applied to patients—should require dedicated training and supervised practice for healthcare professionals, with progressive acquisition of skills needed to manage stress-laden perioperative situations. This approach represents a plausible, and in many respects essential, strategy to be integrated into contemporary perioperative pathways as a “stress vaccine.”

Importantly, although these interventions were not originally designed to enhance resilience per se, they indirectly target psychological constructs—such as catastrophizing, emotional dysregulation, loss of control, and reduced self-efficacy—that are closely linked to diminished resilience. In this light, they offer a valuable proof of concept: if perioperative care can act on the very psychological vulnerabilities that undermine resilience, it may become possible to proactively strengthen patients’ adaptive capacities and ultimately improve recovery trajectories. This perspective supports the hypothesis that enhancing resilience—even through non-specific methods—could represent a clinically meaningful pathway toward more robust, patient-centered surgical outcomes.

### 6.2. Prehabilitation and Multimodal Optimization

Recent studies have shown that prehabilitation and multimodal optimization are now foundational components of the “enhanced recovery after surgery” (ERAS) paradigm [111,112]. This paradigm involves an intentional process of preoperative “conditioning” comprising three complementary dimensions: physical (aerobic training and respiratory exercises), nutritional, and psychological (motivational interviewing, biopsychosocial pain management, clinical hypnosis, relaxation techniques, mindfulness, and sleep optimization). The overarching objective of this complex intervention is to improve surgical fitness, including by reducing vulnerability to perioperative stressors.

Current research indicates that adherence to multimodal programs can translate into clinically meaningful benefits when integrated as a component of perioperative care, as multimodal interventions act on several interdependent physiological domains—namely inflammation, catabolism, cardiorespiratory capacity, and adherence to treatment. Moreover, investigators have shown that such programs may reduce adverse events and hospital length of stay, particularly in major surgical procedures [113].

Meta-analyses of high-intensity preoperative exercise protocols demonstrate substantial increases in cardiorespiratory fitness within only a few weeks and, in several studies, reductions in postoperative complications compared with standard care. Effects on length of stay are more variable but tend to be greater when the program includes well-structured exercise components [114]. Improvements in cardiorespiratory fitness and oxygen consumption may emerge as early as 3–4 weeks with high-density protocols—an essential feature when the preoperative window is short [114]. These syntheses are complemented by pragmatic trials which, despite heterogeneity in clinical outcomes, consistently demonstrate the feasibility and physiological impact of prehabilitation. For example, in a JAMA Surgery study of frail patients undergoing colorectal surgery, a multimodal program (exercise, nutrition, and psychological support) initiated before surgery did not reduce 30-day postoperative complications relative to well-structured postoperative rehabilitation within an ERAS pathway—likely owing to the exclusive inclusion of frail patients [115].

In parallel, cumulative analyses show that preoperative respiratory physiotherapy is associated with reduced hospital stay and may influence respiratory outcomes in high-risk settings. These findings support the selective incorporation of respiratory training into protocols for procedures with elevated respiratory risk [116].

Nutrition is a cross-cutting determinant of postoperative outcome because it modulates the catabolic, and indirectly, immune response to surgery. European guidelines recommend systematic nutritional screening and the initiation of oral nutritional supplements when caloric intake is inadequate, irrespective of baseline nutritional status. Immunonutrition formulas may be considered and depending on risk profile and time available before surgery, enteral nutritional support—or, when not feasible, parenteral support—may be warranted [113]. Real-world data from the Prehab4Cancer program show that many oncology patients entering prehabilitation are at risk of malnutrition, and that a structured pathway allows a proportion of them to improve risk indicators and maintain stable weight following surgery—an essential factor for preserving muscle mass and strength [117].

In colorectal surgery, cumulative analyses indicate that the use of probiotics or probiotic–prebiotic combinations in the perioperative period may reduce postoperative infections, with favorable trends for pneumonia and urinary tract infections [118].

Beyond CBT and mindfulness-based interventions, the psychological dimension includes tools that facilitate stress management and the correction of dysfunctional beliefs about pain and recovery. In a landmark study of high-risk patients undergoing major abdominal surgery, personalized prehabilitation included motivational interviewing, high-intensity cycle ergometer resistance training, and promotion of daily physical activity. This program increased aerobic capacity before surgery and reduced postoperative complications by 51%. These findings indicate that motivation is a decisive factor in achieving optimal response to prehabilitation [119].

Pain Neuroscience Education (PNE) aims to restructure maladaptive cognitions and emotions—such as fear of movement and catastrophizing—and to strengthen coping strategies. When delivered perioperatively, PNE has demonstrated clinical superiority over standard “biomedical” education, with signals of sustained benefit at follow-up [120,121].

Clinical hypnosis, based on suggestive communication, has been shown—particularly in breast surgery—to reduce preoperative anxiety even when administered in brief sessions immediately before anesthesia, and multiple studies report reductions in postoperative pain as well [122,123]. Recent data on hypnosis used as a sedation technique in oncologic breast surgery also suggest reductions in early inflammatory markers, such as the neutrophil-to-lymphocyte ratio and C-reactive protein, although confirmation in randomized trials is required [124].

Regarding sleep hygiene, evidence indicates associations between sleep disruption and poorer stress, pain, and cognitive outcomes. A 2024 systematic review reported that sleep is modifiable preoperatively using pharmacological strategies and brief sessions of cognitive-behavioral therapy for insomnia (CBT-I) [125].

A critical challenge for integrating these strategies into contemporary clinical practice is determining the appropriate dose of psychological, nutritional, and physical prehabilitation—namely, which patients benefit most, for how long, at what intensity, with what timing, and how best to embed these interventions within the entire perioperative care pathway.

Taken together, these findings broaden the conceptual boundaries of perioperative optimization by suggesting that elements traditionally considered “physical” or “physiological”—such as nutrition, respiratory training, or cardiorespiratory conditioning—may also contribute to strengthening resilience. In other words, resilience should not be viewed exclusively as a psychological construct, but rather as a multidimensional capacity that can be reinforced through parallel interventions targeting both mental and physical domains. Adopting this broader framework allows us to interpret prehabilitation not only as risk reduction, but as a means to enhance the patient’s overall resilience profile in preparation for surgical stress. This integrative perspective may ultimately support the development of more comprehensive, patient-centered perioperative pathways aimed at fortifying adaptive resources across multiple levels of functioning.

### 6.3. Pharmacological and Biological Adjuncts

The goal of pharmacological and biological interventions is to dampen autonomic nervous system stress, reduce inflammation and pain, and—where microbiota-directed strategies are used—modulate the bidirectional signaling between the gut and the brain. Within this framework, antidepressants, gabapentinoids (pregabalin and gabapentin), beta-adrenergic receptor blockers, anti-inflammatory agents, and psychobiotic interventions play important roles in constructing an integrated perioperative strategy. The overarching aim is to “cool down” the physiological stress response to surgery, reduce opioid exposure, and prevent postoperative complications.

Because SSRIs are among the most commonly used chronic medications requiring management in the perioperative period, clinicians must balance psychiatric stability with coagulation risk and drug–drug interactions. Several lines of evidence suggest a modest increase in bleeding risk due to reduced platelet aggregation. The magnitude of risk varies according to the type of surgery and co-exposures (antiplatelet agents, anticoagulants, NSAIDs) [126,127,128,129,130].

In plastic and reconstructive surgery, a 2025 systematic review evaluated the association between SSRIs and perioperative bleeding. Five retrospective cohorts were included, mostly involving breast and craniofacial procedures. Reported bleeding rates ranged from 1.9% to 2.6%. In breast surgery, there was an increased risk of reoperation for hematoma, whereas a Danish cohort study found no elevated bleeding risk. In craniofacial procedures, two U.S. studies did not show significant differences in hemorrhagic events, although power was limited. Overall, the authors concluded that the absolute risk of clinically meaningful hemorrhagic complications is low across many procedures (including craniofacial), with a slight increase in reoperation for hematoma in breast surgery and no evidence of life-threatening bleeding [131].

In general, continuing SSRIs perioperatively is often appropriate, except in surgeries with intrinsically very high bleeding risk (e.g., neurosurgery, extensive hepatic resections, major otolaryngologic surgery) or when combined with antithrombotic agents that cannot be modified.

The rationale for duloxetine use is to enhance descending inhibitory pain pathways and modulate perioperative hyperalgesia. In hip and knee arthroplasty, cumulative analyses published between 2023 and 2024 show reductions in opioid consumption and improvements in pain, particularly during the early postoperative period and up to 2–3 weeks, with an overall acceptable safety profile. A 2024 meta-analysis of eight randomized trials (695 patients) demonstrated a standardized reduction in opioid use and analgesic benefit within three weeks [132]. A 2023 systematic review and meta-analysis confirmed lower pain scores on postoperative days 1, 3, 7, and 14, at six weeks, and at three months, as well as reduced opioid use at 48–72 hours, although with heterogeneity and small samples [133]. In a triple-blind randomized trial of knee arthroplasty, duloxetine 60 mg/day initiated on the day of surgery for 14 days reduced opioid consumption by 29% without increasing pain, using a non-inferiority design centered on pain outcomes [134]. In hip arthroplasty, duloxetine 60 mg/day administered from two days before to 14 days after surgery reduced pain scores during the first three weeks and decreased morphine use at 48 h, though without surpassing the minimal clinically important difference for pain during movement [135]. A prior knee arthroplasty study did not reduce the primary pain endpoint but was associated with lower opioid use [136]. In patients with signs of central sensitization undergoing knee arthroplasty, 30 mg/day starting one day before surgery and continued for six weeks improved pain (between weeks 2 and 12) and quality of recovery (at two weeks) [137]. A second “pre-emptive” trial with duloxetine 30 mg/day confirmed pain reduction up to six weeks [138].

A large systematic review and meta-analysis of 281 randomized trials (24,682 participants) found no clinically meaningful benefit of gabapentinoids for acute postoperative pain. Instead, gabapentinoids were associated with more dizziness and visual disturbances, but with less nausea and vomiting compared with control [139]. In joint arthroplasty, a 2025 meta-analysis focusing on pregabalin showed reductions in pain with movement at 24, 48, and 72 h and lower opioid use at the same time points. However, increased sedation and visual disturbances were observed. Improvements in range of motion at 72 h and greater efficacy when the drug was started more than 24 h preoperatively were also reported [140]. Among historical trials, a knee arthroplasty protocol using 300 mg pregabalin preoperatively followed by 150–50 mg twice daily for 14 days reduced neuropathic pain at three and six months, lowered opioid consumption, and improved knee flexion during the first 30 days—at the cost of more frequent sedation and confusion immediately postoperatively [141].

In hip and knee arthroplasty, perioperative COX-2 inhibitors reduce pain during the first 24–72 h and decrease opioid requirements in the first 24–48 h. On average, they are also associated with less nausea and vomiting, without signals of serious complications in available data [142].

Regarding hemostatic safety, a large review found no increase in hematoma, surgical reintervention, or transfusion among patients receiving NSAIDs compared with those not receiving them. Thus, in the absence of personal contraindications, their inclusion in multimodal postoperative analgesia is justified [143].

In colorectal surgery, randomized and observational studies suggest that perioperative administration of probiotics or probiotic–prebiotic combinations reduces the risk of postoperative infections at 30 days, with favorable trends for pneumonia and urinary tract infections [118]. In major hepatobiliary surgery, fewer postoperative infections and approximately three fewer days of antibiotic therapy have been observed, although no clear differences emerged in ICU stay or total hospital stay [144]. On the gut–brain axis side, a randomized study of a four-week “psychobiotic diet” in healthy adults demonstrated reductions in perceived stress—particularly among highly adherent participants—with coherent changes in metabolomic profiles and microbiota composition [145].

In conclusion, psychiatric pharmacotherapy could be viewed not solely as a treatment for psychiatric comorbidity, but as a potential adjunct to enhance overall surgical fitness and psychological well-being. By stabilizing mood, improving cognitive-emotional regulation, and attenuating stress responsivity, pharmacological strategies may contribute to a more adaptive perception of hospitalization and facilitate a stronger, more resilient engagement with the perioperative journey. Although this hypothesis remains largely unexplored, it offers a compelling rationale for integrating psychotropic treatment within broader resilience-oriented perioperative frameworks—moving beyond symptom management toward the proactive reinforcement of patients’ adaptive capacities.

**Figure 2:**
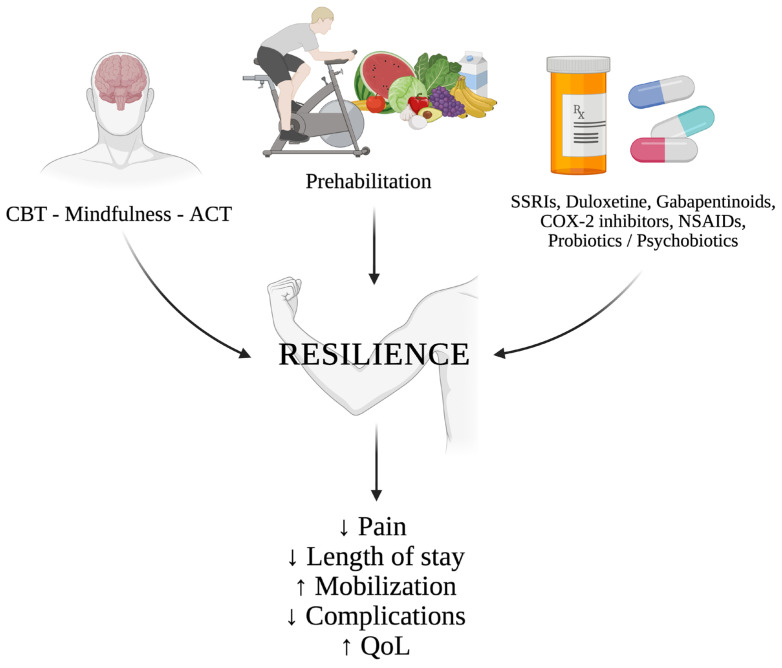
Core perioperative strategies that enhance psychological resilience. Psychological and behavioral interventions—namely Cognitive Behavioral Therapy (CBT), Mindfulness-Based Interventions (MBIs), and Acceptance and Commitment Therapy (ACT)—together with multimodal prehabilitation (exercise and nutrition) and pharmacological/biological adjuncts, including Selective Serotonin Reuptake Inhibitors (SSRIs), duloxetine, gabapentinoids, Cyclooxygenase-2 (COX-2) inhibitors, Non-Steroidal Anti-Inflammatory Drugs (NSAIDs), and probiotics/psychobiotics, converge to strengthen resilience prior to surgery. Increased RES is associated with reduced pain, shorter Length of Stay (LOS), improved mobilization, fewer complications, and higher Quality of Life (QoL). Figure created with BioRender.com.

As illustrated in Figure 2, the integration of psychological, behavioral, and pharmacological strategies within perioperative care may enhance resilience and improve recovery trajectories.

## 7. Clinical Implications

### 7.1. Screening for Resilience as Part of Preoperative Risk Assessment

Screening surgical patients for psychological resilience is an emerging strategy to enhance preoperative risk assessment and personalize perioperative care. Resilience is increasingly recognized as a potentially modifiable perioperative risk factor. Patients with low resilience often struggle more with surgical stress and recovery, whereas higher resilience (or mental strength) has been linked to better postoperative outcomes [48]. Incorporating resilience into preoperative screening could help identify at-risk patients early and enable targeted interventions to improve their surgical trajectory.

Several brief, validated questionnaires can assess resilience in adults prior to surgery. A recent scoping review of 74 studies identified seven tools used in surgical populations [48]. The most common are the Brief Resilience Scale (BRS) and the Connor–Davidson Resilience Scale (CD-RISC). The BRS is a six-item self-report tool evaluating one’s ability to “bounce back” from stress [37], while the CD-RISC (available in 25-, 10-, or 2-item versions) measures traits such as perseverance and optimism [36]. These instruments are concise, easy to administer, and validated in adult populations, making them suitable for preoperative clinics.

Growing data suggest that preoperative resilience scores correlate with key surgical outcomes. Higher resilience has consistently been associated with improved patient-reported experiences—better postoperative quality of life, higher satisfaction, and lower pain levels. Some studies have also linked resilience to objective recovery measures. For example, in patients undergoing ACL reconstruction, low preoperative BRS scores were associated with worse knee function and slower return to sport [146]. In older adults facing major surgery, mental resilience may even affect survival and functional recovery: in a multicenter hip-fracture cohort, patients with higher BRS scores had lower odds of death or loss of independent ambulation within 60 days [98]. These findings underscore that psychological resilience, though intangible, can meaningfully influence physical recovery.

Integrating resilience screening into preoperative assessment could enhance risk stratification beyond conventional metrics. It provides insights not captured by standard risk tools. For instance, among lung transplant candidates, 16% scored more than one standard deviation below the population norm on the 10-item CD-RISC; this subgroup had more than twice the risk of pre-transplant mortality or delisting. Notably, resilience scores in that study did not correlate with standard psychosocial evaluations, highlighting the added value of resilience assessment [147]. Identifying low-resilience patients before surgery allows clinicians to anticipate potential challenges (e.g., poor coping, prolonged recovery) and implement supportive strategies such as psychological prehabilitation, stress-management training, or counseling.

Although tools like the BRS and CD-RISC are validated and increasingly used in research, their routine clinical use remains experimental. No universally accepted “resilience score” is yet integrated into surgical risk calculators. However, experts advocate for more systematic assessment of resilience across diverse surgical populations [48]. Some propose coupling resilience with frailty to achieve a more holistic risk profile; indeed, combining physical frailty with psychological resilience may better predict outcomes than age alone. A recent systematic review found evidence linking preoperative resilience to outcomes still inconclusive but emphasized the promise of the concept and the need for further study [48]. Resilience screening remains an evolving field, but early evidence suggests it could become a valuable addition to perioperative risk assessment—one that helps tailor interventions and improves surgical recovery by addressing patients’ mental preparedness.

### 7.2. Incorporation into Enhanced Recovery After Surgery (ERAS) Pathways

Higher levels of resilience have been associated with better postoperative quality of life, lower pain scores, and fewer psychological symptoms, suggesting that resilient patients cope more effectively with surgical stress [148]. Within the framework of Enhanced Recovery After Surgery (ERAS) protocols [111] —which aim to minimize surgical stress and accelerate recovery—integrating resilience screening and resilience-building interventions represents a logical extension of patient optimization. Early evidence indicates that preoperative resilience influences outcomes: higher resilience was consistently linked to better patient-experience domains, including quality of life, satisfaction, mental health, and pain control, as well as some functional outcomes after surgery [48]. Although data connecting resilience to “hard” endpoints such as complication rates or length of stay remain limited, resilience has emerged as a modifiable factor worthy of systematic consideration in ERAS pathways. These observations have prompted calls for routine resilience assessment in perioperative care to identify patients who may benefit from targeted support [149].

Some ERAS-oriented programs have already incorporated resilience-enhancing strategies into their multimodal design. Prehabilitation—a cornerstone of ERAS—intrinsically fosters resilience by improving both physical fitness and psychological readiness for surgery [150]. Structured exercise in prehabilitation not only increases cardiopulmonary reserve but also enhances emotional well-being, helping patients better manage surgical stress. Many prehabilitation protocols include dedicated psychosocial components such as relaxation training, stress management, or counseling sessions to strengthen mental resilience. A systematic review confirmed that adding psychological interventions (e.g., cognitive-behavioral therapy or mindfulness-based stress reduction) can reduce preoperative anxiety and improve adherence to ERAS activities [149]. These approaches also improved patient satisfaction and reduced postoperative stress responses, aligning perfectly with ERAS’s holistic recovery philosophy.

Prospective clinical data reinforce this trend. Wang et al. evaluated a prehabilitation program—combining exercise, nutritional counseling, and anxiety-reduction training—before colorectal surgery within a standard ERAS framework [150]. The intervention group achieved greater functional capacity at surgery and significantly lower anxiety scores than controls. Although these improvements did not translate into reduced complications or hospital stay, the trial demonstrated that resilience-building measures can enhance patient-centered outcomes without adverse effects, and are feasible even in resource-limited contexts.

Beyond individual studies, conceptual frameworks are emerging to formally embed resilience into ERAS pathways. One approach involves systematic resilience screening during preoperative assessment. Using validated tools such as the Brief Resilience Scale (BRS) or the Connor–Davidson Resilience Scale (CD-RISC) [48], surgical teams can identify low-resilience patients and tailor interventions accordingly—such as psychological support, stress-reduction programs, or enhanced postoperative follow-up [151]. A prospective cohort study in gynecologic surgery exemplified this model: women with higher resilience reported less pain and better recovery one-year post-surgery, leading the authors to advocate preoperative identification and targeted psychosocial support for low-resilience patients [6].

An innovative alternative involves integrating resilience monitoring directly into ERAS via patient-engagement tools. Graffigna and colleagues proposed the Patient Health Engagement (PHE) model to track engagement levels reflecting psychological resilience [152]. In an Italian ERAS registry for thoracic surgery, the PHE scale was embedded to capture real-time resilience data during the perioperative journey. This pilot allowed clinicians to detect patients struggling with surgical stress and to deploy timely supportive measures, operationalizing a theoretical resilience framework in clinical practice. Early reports describe this as a cultural shift—placing the patient’s psychological state on par with traditional clinical metrics—and suggest that correlating resilience trajectories with outcomes could provide valuable insights for real-world ERAS optimization [152].

Embedding resilience into ERAS pathways represents a decisive move toward personalized, patient-centered perioperative care. In practice, this means that beyond optimizing physiological parameters, care teams actively strengthen patients’ mental and emotional capacity to endure surgical stress. The potential benefits are twofold: patients experience improved well-being, with less anxiety, better pain control, and greater satisfaction; enhanced resilience may translate into smoother recoveries—quicker mobilization, stronger adherence to ERAS components, and possibly fewer complications—although evidence for these “hard” outcomes remains under investigation [149].

Importantly, integrating resilience does not replace existing ERAS elements but complements them. It synergizes with interventions such as early mobilization and multimodal analgesia by ensuring that patients are psychologically prepared to engage fully in recovery. Practical measures may include multidisciplinary prehabilitation teams incorporating psychologists or trained counselors, routine use of resilience or stress-assessment questionnaires to personalize preoperative education, and postoperative debriefings or coping-skills sessions as part of discharge planning. These low-risk initiatives fit naturally within ERAS’s multimodal ethos. They embody the idea of “prehabilitation of the mind,” extending perioperative optimization into the psychosocial domain [150].

Future translational research should focus on standardizing resilience measurement, refining intervention strategies, and clarifying their impact on surgical outcomes. Ongoing trials are exploring whether resilience training—through mindfulness, cognitive reframing, or digital coaching—can improve both subjective and objective recovery indices [153]. As evidence accrues, formal guidelines may soon recommend routine resilience screening in ERAS, similar to current use of frailty indices. In summary, incorporating resilience into ERAS pathways acknowledges psychological fortitude as a modifiable determinant of recovery. Early resilience-based interventions have improved patient-experience metrics, while conceptual models offer feasible blueprints for broader implementation [154]. Ultimately, this evolution could humanize surgical care and usher in a new era of ERAS innovation centered on the resilient patient.

The main practical implications emerging from the available evidence are summarized in Table 1.

## 8. Conclusions and Future Directions

Resilience in the perioperative setting represents a complex and multidimensional construct, shaped by psychological traits, biological systems, and social determinants. The existing data suggest that resilience may serve as a unifying framework to interpret interindividual variability in recovery and to identify patients who are more vulnerable to surgical stress.

At present, the integration of resilience into perioperative medicine should not be viewed as a definitive solution, but rather as a necessary shift in perspective. Resilience ought to be considered a core element of patient assessment—both for early identification of at-risk individuals and for the development of targeted interventions designed to strengthen adaptive resources before and after surgery. Achieving this goal requires rigorous methodological development. Existing self-report resilience scales, while widely used, remain anchored in trait-based models and lack validation in surgical cohorts; moreover, they often fail to capture domain-specific variability or the dynamic, time-sensitive nature of resilience during recovery. As such, adaptation of current instruments or the creation of new perioperatively focused measures is necessary, ideally incorporating repeated assessments to reflect fluctuations across the surgical timeline. Importantly, resilience should not remain the sole domain of psychiatry or psychology. Surgeons, anesthesiologists, and perioperative care teams need to regard it as a clinically meaningful and modifiable determinant of surgical outcomes—one that can be addressed proactively through prehabilitation protocols, stress-modulation strategies, and structured biopsychosocial interventions. Some patients may demonstrate high resilience in daily life yet struggle specifically in the surgical context due to heightened uncertainty, acute pain, loss of autonomy, etc. This distinction underscores the need for context-sensitive, precision-oriented assessment tools that capture how patients mobilize adaptive capacity within the unique demands of surgery. Looking ahead, the perioperative setting could serve as a powerful natural laboratory for advancing resilience research through integrative models combining psychometric measures, laboratory assays, and neuroimaging. Dedicated programs aimed at enhancing resilience should be developed and tested prospectively, acknowledging that resilience is modifiable and dynamically responsive to intervention. By embedding resilience, both conceptually and operationally, into perioperative care, we may progress toward a more holistic, personalized, and psychologically informed model of surgical medicine, one that integrates biological, psychological, and social domains to optimize patient outcomes.

## Figures and Tables

**Table 1:** Clinical Implications of Resilience Assessment and Integration in Perioperative and ERAS Pathways.

Domain	Clinical Rationale	Practical Implementation	Expected Clinical Impact
**Preoperative Risk** **Stratification**	Low resilience is associated with poorer psychological adjustment, increased pain perception, and slower functional recovery.	Administer brief validated tools (BRS, CD-RISC) during preoperative evaluation.	Early identification of vulnerable patients; more personalized perioperative planning.
**Frailty and Multidimensional Risk Profiling**	Resilience complements physical frailty measures by capturing psychological adaptability.	Combine resilience screening with frailty indices and standard surgical risk calculators.	More holistic prediction of postoperative trajectories beyond age and comorbidity alone.
**Psychological ** **Prehabilitation**	Resilience is potentially modifiable before surgery.	Implement structured prehabilitation including stress-management training, CBT-based interventions, mindfulness, or counseling.	Reduced preoperative anxiety; improved coping; better adherence to ERAS components.
**ERAS Integration**	ERAS aims to minimize stress and optimize recovery; resilience influences stress response.	Embed resilience assessment into ERAS pathways; tailor education and support accordingly.	Improved patient-reported outcomes (quality of life, satisfaction, pain control).
**Targeted Support for High-Risk Patients**	Low-resilience patients may have higher risk of delayed recovery or disengagement.	Provide intensified follow-up, psychological support, and discharge planning.	Smoother postoperative recovery; potential reduction in readmissions and prolonged recovery.
**Patient Engagement and Monitoring**	Resilience may fluctuate across the perioperative journey.	Use engagement scales (e.g., PHE model) or digital tools to monitor psychological adaptation longitudinally.	Early detection of distress; timely intervention during recovery.
**Future Guideline** **Development**	Growing evidence supports resilience as a modifiable determinant of recovery.	Standardize measurement tools and integrate resilience into clinical protocols.	Movement toward personalized, psychologically informed surgical care.

*Notes*: BRS (Brief Resilience Scale), CBT (Cognitive-Behavioral Therapy), CD-RISC (Connor–Davidson Resilience Scale), ERAS (Enhanced Recovery After Surgery), PHE (Patient Health Engagement).

## Data Availability

No new data were created or analyzed in this study. Data sharing is not applicable to this article.

## Abbreviations

The following abbreviations are used in this manuscript:

ACT	Acceptance and Commitment Therapy
ACTH	Adrenocorticotropic Hormone
ACL	Anterior Cruciate Ligament
ANS	Autonomic Nervous System
aOR	Adjusted Odds Ratio
BRS	Brief Resilience Scale
CBT	Cognitive Behavioral Therapy
CBT-I	Cognitive Behavioral Therapy for Insomnia
CD-RISC	Connor–Davidson Resilience Scale
CI	Confidence Interval
CRF	Corticotropin-Releasing Factor
CRP	C-reactive Protein
ERAS	Enhanced Recovery After Surgery
GR	Glucocorticoid Receptor
HRV	Heart Rate Variability
HPA	Hypothalamic–Pituitary–Adrenal (axis)
ICU	Intensive Care Unit
IL	Interleukin
MDD	Major Depressive Disorder
MR	Mineralocorticoid Receptor
NSAIDs	Non-Steroidal Anti-Inflammatory Drugs
PHE	Patient Health Engagement
PNE	Pain Neuroscience Education
PNS	Parasympathetic Nervous System
PTSD	Post-Traumatic Stress Disorder
RSA	Resilience Scale for Adults
SAM	Sympathetic–Adrenal–Medullary (axis)
SNS	Sympathetic Nervous System
SSRIs	Selective Serotonin Reuptake Inhibitors
TNF-α	Tumor Necrosis Factor Alpha
VR	Virtual Reality
WHO	World Health Organization
